# Path model of antenatal stress and depressive symptoms among Chinese primipara in late pregnancy

**DOI:** 10.1186/s12884-016-0972-2

**Published:** 2016-07-21

**Authors:** Yingtao Li, Yingchun Zeng, Wei Zhu, Ying Cui, Jie Li

**Affiliations:** Department of Obstetrics, The Third Affiliated Hospital of Guangzhou Medical University, Guangzhou, China; Department of Nursing, Shandong Medical College, Jinan, China; Department of Psychiatry, The Third Affiliated Hospital of Guangzhou Medical University, Guangzhou, China; Guangzhou Brain Hospital, Guangzhou Medical University, Guangzhou, China

**Keywords:** Antenatal stress, Depressive symptoms, Chinese women, Path analysis, Conceptual model

## Abstract

**Background:**

Antenatal maternal mental health problems have numerous consequences for the well-being of both mother and child. This study aimed to test and construct a pertinent model of antenatal depressive symptoms within the conceptual framework of a stress process model.

**Methods:**

This study utilized a cross-sectional study design. Inclusion criteria: participants were adult women (18 years or older) having a healthy pregnancy, in their third trimester (the mean weeks gestation was 34.71). Data collection: depressive and anxiety symptoms were measured by Zung’s Self-rating Depressive and Anxiety Scale, stress was measured by Pregnancy-related Pressure Scale, social support and coping strategies were measured by Social Support Rating Scale and Simplified Coping Style Questionnaire, respectively. Analysis: path analysis was applied to examine the hypothesized causal paths between study variables.

**Results:**

A total of 292 subjects were enrolled. The final testing model showed good fit, with normed *χ*^2^ = 32.317, *p* = 0.061, CFI = 0.961, TLI = 0.917, IFI = 0.964, NFI = 0.900, RMSEA = 0.042. This path model supported the proposed model within the theoretical framework of the stress process model. Pregnancy-related stress, financial strain and active coping have both direct and indirect effects on depressive symptoms. Psychological preparedness for delivery, social support and anxiety levels have direct effects on antenatal depressive symptoms. Good preparedness for delivery could reduce depressive symptoms, while higher levels of anxiety could significantly increase depressive symptoms. Additionally, there were indirect effects of miscarriage history, irregular menstruation, partner relationship and passive coping with depressive symptoms.

**Conclusion:**

The empirical support from this study has enriched theories on the determinants of depressive symptoms among Chinese primipara, and could facilitate the formulation of appropriate interventions for reducing antenatal depressive symptoms, and enhancing the mental health of pregnant women.

## Background

Antenatal maternal mental health problems have numerous consequences for the well-being of both mother and child [1, 2]. While pregnancy is a joyful event for most women [3], perceived stress and depressive symptoms during pregnancy have paid the significant role in adverse birth outcomes as well as maternal well-being [4]. Research reported that half of all individuals with major depressive disorders experience significant depressive symptoms before the first identified episode [5], and prenatal maternal stress is also common and as high as 36.1 % women experienced certain level of stress during pregnancy [6].

Peripartum depression is a potentially devastating disorder that can have devastating consequences for the affected woman. Previous studies indicate that depression during pregnancy has been linked to poor childbirth outcomes, such as spontaneous preterm delivery and low birth weight [7, 8]. The symptoms of maternal depression can lead to the mother rejecting her role of caring for her child [9]. Most severely, the risk of maternal suicide is high among depressed perinatal women [1, 10]. Perinatal mental problems are also linked to increased risk of psychological and developmental disturbances in children [11]. Research evidence shows that maternal depressive symptoms could have a negative impact on fetal immune development [12], as well as a high risk of emotional and behavioral problems and cognitive development delay [13, 14].

Profound changes during the perinatal period can trigger stress in pregnant women [15]. Stress during pregnancy has been linked to increased incidence of antenatal depressive or anxiety symptoms [16, 17]. Psychosocial stress theory identifies social support as a protective factor against depressive symptoms during pregnancy [18]. While there has been increased research-expanded awareness in the last decade of the importance of antenatal maternal mental health among health professionals [1], empirical research does not elucidate the causal direction of the relationship between influencing factors and antenatal mental health outcomes [3].

Psychological science on stress, stress management and consequences emphasizes mechanisms underlying relationships between variables and health outcomes [19, 20]. In particular, research on pregnancy and birth is a unique research context, which concerns women at a critical time in their lives, a time that is infused with significance for their health and well-being and that of their children, spouse, and families [20]. Moreover, Kingston et al. [6] also highlights the importance of incorporating multiple measures of stress and modeling the stress process within a theoretical framework for research on psychosocial risk assessment in pregnancy. Hence, the study of antenatal stress and depressive symptoms under the stress process model is highly relevant to deriving more useful theory and research on pregnancy and maternal health outcomes.

**Fig. 1:**
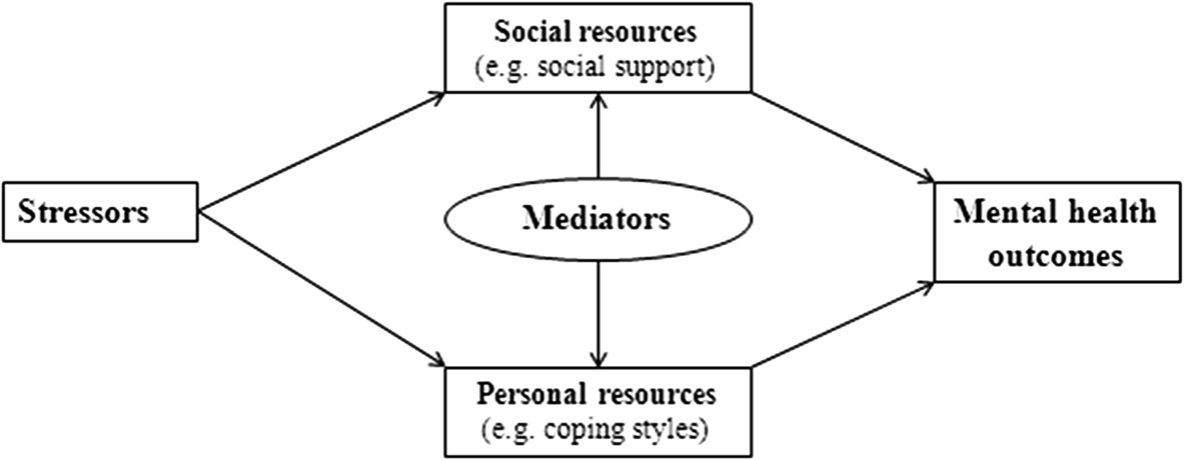
Stress process model

There are three fundamental concepts that form the stress process: stressors, mediators, and stress outcomes [21]. Stressors can be external or internal factors that challenge pregnant women to adapt or change. Mediators are the social or personal resources that attenuate the effects of the stressors. By accounting for the mediators, stress outcomes are psychological or physiological conditions resulting from exposure to stressors [3]. In this study, clinical and socio-demographic characteristics and profound changes, such as physical and psychological changes during pregnancy, were viewed stressors; social support and personal resources for coping strategies can be taken as mediators; and antenatal depressive symptoms represent psychological conditions of stress outcomes. The conceptual model guiding this study is illustrated in Fig. 1.

Accordingly, the aims of this study were to test the stress process model among Chinese primipara in late pregnancy. It was hypothesized that stressors would be mitigated by these women’s social and personal resources. Certainly, perceived stress would be influenced by participants’ clinical and socio-demographic characteristics. In addition, Chinese women endowed with both social support and positive coping strategies would perceive lower levels of stress during pregnancy, and hence would experience reduced depressive symptoms. Understanding how these factors interact with one another could support health professionals in developing effective interventions to help affected women.

## Methods

### Participants

Participants were a sample of primiparous mothers recruited from antenatal clinics in a maternal and child hospital in South China. The antenatal phase comprised 292 participants consecutively recruited over 3 months. Eligible criteria for participants were being adult women (18 years or older) experiencing a healthy pregnancy, in their third trimester. Multiparous women and women in the first and second trimester, women with a mental disorder or having a high-risk pregnancy due to maternal (e.g., cardiovascular disease) conditions were excluded.

### Measures

#### Depressive symptom measure

Antenatal depressive symptoms were measured by the Self-rating Depression Scale (SDS). The SDS was originally developed by Zung [22], and is one of the most widely used self-rating measures for clinicians to identify depressive symptoms in adults [23]. The SDS is a 20-item Likert-style (4-point) rating scale for depression and anxiety, with a theoretical score range extending from 20 to 80. The original total score plus 1.25 will be the standardized score (<50 as normal; 50–59 as mild depressive cases; 60–69 as moderate depressive cases; and ≥70 as severe depressive cases) [23]. The Chinese version of SDS is widely used among Chinese women during pregnancy [24, 25]. The internal consistency by Cronbach’s alpha of SDS in this study was 0.91.

#### Anxiety symptom measure

Antenatal anxiety symptoms were measured by Self-rating Anxiety Scale (SAS). The SAS was originally developed by Zung [26], and is one of the most widely used self-rating measures for clinicians to identify anxiety symptoms in adults [23]. The SAS is a 20-item Likert-style (4-point) rating scale for anxiety, with a theoretical score range extending from 20 to 80. The original total score plus 1.25 will be the “Anxiety Index” score (<50 as normal; 50 - 59 as mild depressive cases; 60 - 69 as moderate depressive cases; and ≥70 as severe depressive cases) [23]. The Chinese version of SAS is also widely used among Chinese women during pregnancy [24]. The internal consistency by Cronbach’s alpha of SAS in this study was 0.89.

#### Antenatal stress measure

Antenatal stress was measured by both Pregnancy Pressure Scale (PPS) and self-rating anxiety scale (SAS). The PPS consists of 30 items [27]. Responses to each question ranged from 0 (never) to 4 (very often). All items were added to a total pressure score. Higher scores indicate a high pressure level. The PPS has demonstrated an acceptable reliability among Chinese women [27]. The internal consistency by Cronbach’s alpha of PPS among Chinese pregnant women was 0.92 [25].

#### Coping strategy measure

The Chinese version of Simplified Coping Style Questionnaire (SCSQ) was used to assess participants’ coping tendencies. The SCSQ consists of 20 items with two subscales: “active coping” (12 items) and “passive coping” (8 items) [28]. Active coping emphasizes positive coping characteristics, such as “handling the distressing emotions caused by the problem”. Passive coping emphasizes the characteristics of negative coping, such as “escaping troubles by drinking and smoking” [28]. A higher score for each dimension indicates frequent use of the coping style. Previous research reported good reliability of the Chinese version of SCSQ among Chinese pregnant women [25]. The internal consistency of the SCSQ by Cronbach’s alpha was 0.81 for the active coping subscale and 0.76 for the passive coping subscale in the current study.

#### Social support measure

Social support was measured by Social Support Rating Scale (SSRS). The SSRS was originally developed in China by Xiao [29]. This scale is comprised of 10 items. Higher scores indicate better social support from family, friends and significant others. The SSRS was widely used in assessing social support for Chinese women [24, 25]. The internal consistency of SSRS among Chinese pregnant women was 0.89 by Cronbach’s alpha in the current study.

#### Socio-demographic and clinical measure

Socio-demographic characteristics include age, education level, medical payment type, quality of relationship with spouse, desired fetus gender, and financial concerns. Clinical factors include stage of pregnancy, maternal and fetus health status, antenatal health check, history of miscarriage, menstruation history, fetus health concerns, and psychological preparedness for delivery.

### Data collection and procedure

This study was conducted at outpatient clinic of Obstetrics Department of one hospital at South China. Ethical approval was obtained from the ethics review committee of the studied hospital. Midwives with trained research skills recruited women consecutively during their routine antenatal visit. All women participated on a voluntary basis and gave their written informed consent before data collection. A total of 350 women were approached, there were 315 women joined in this study and completed the questionnaire, and 17 copies of data had high percentages of missing data, so that only 292 subjects were included for data analysis.

### Data analysis

**Fig. 2:**
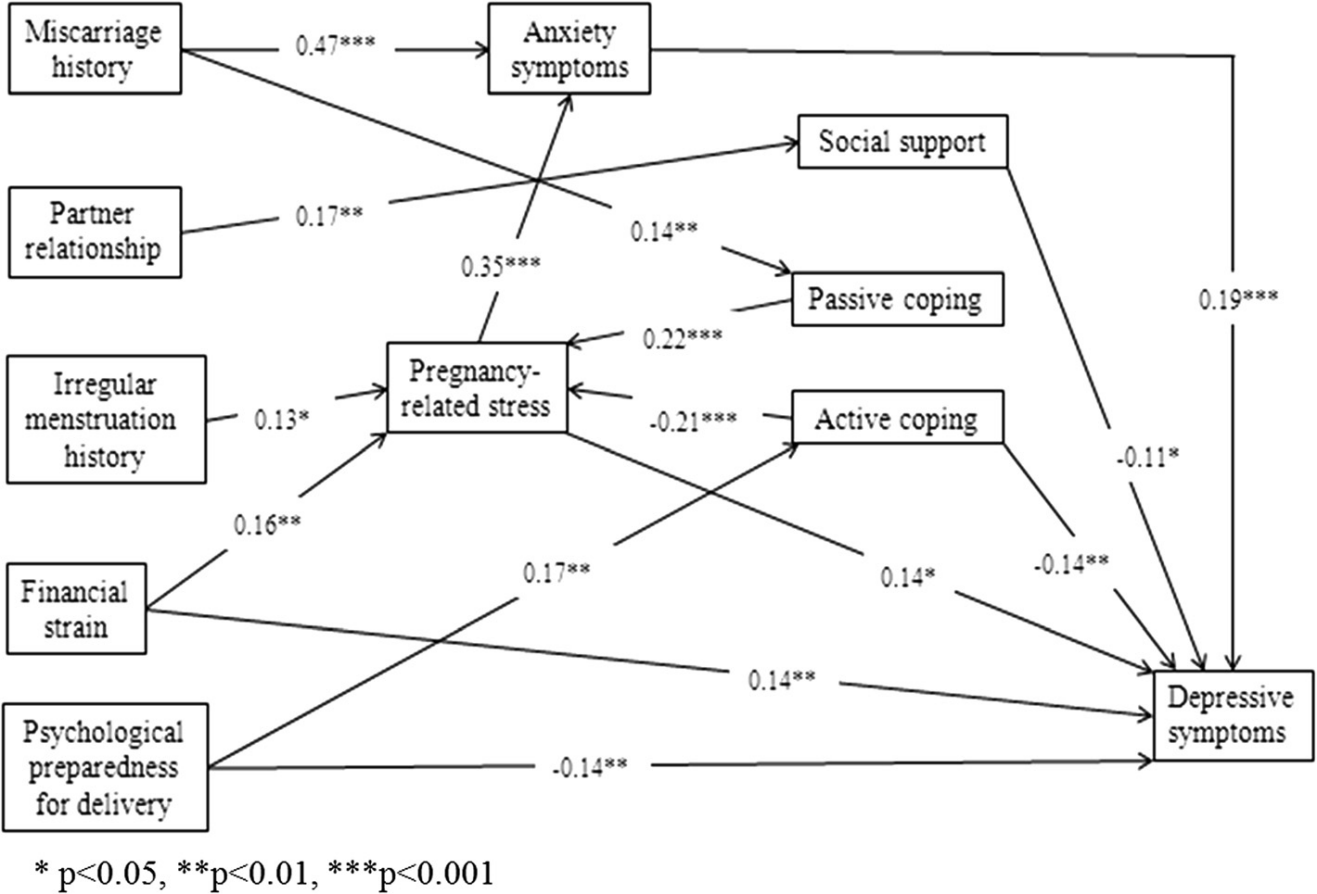
Path model of relationships among stressors, mediators and depressive symptoms

Data were analyzed using the Statistical Package for Social Sciences (SPSS) and Analysis of Moment Structures (AMOS) for Windows, version 20.0. P-values less than 0.05 were regarded as statistically significant. The findings would be summarized by comparison statistics and path analysis. Chi-square or t-tests were used to find differences between non-depressive and depressive women in terms of measured outcomes. Path analytic approach was used to portray the relationship of different variables with the outcome variable (e.g. depressive symptoms) and to test the hypothesized causal paths between study variables. Prior to performing the path analysis through multiple regression analysis, only statistically significant paths (*P* < 0.05) were used to build up an initial path model based on the hypothesized model in Fig. 1. Then, this initial path model was modified by adding plausible paths with the use of modification indices by computing direct or indirect effects and timed to obtain the final path model illustrated in Fig. 2. Standard beta weights were used to represent path coefficients. The overall model fit was examined using goodness-of-fit indices. A non-significant Chi-square value (*P* > 0.05), normed fit index (NFI ≥ 0.90), incremental fit index (IFI ≥ 0.90), Tucker-Lewis index (TLI ≥ 0.90), comparative fit index (CFI ≥ 0.90), and root mean square error of approximation (RMSEA ≤ 0.08) [30].

## Results

As shown in Table 1, there were significant differences in terms of age, number of miscarriages, maternal health status, partner relationship, delivery preparedness, financial strain, antenatal stress, anxiety levels, active coping and total social support between normal women and women with depressive symptoms (all *P* values < 0.05). In other words, women with depressive symptoms more often showed the following characteristics: younger in age, a more frequent history of miscarriages, irregular menstruation history, higher antenatal stress, poor preparedness for delivery, financial strain, less tendency toward active coping and lower levels of social support.

**Table 1:** Comparisons of demographic and clinical features of primipara

Variables	Without depressive symptoms (SDS < 50) *n* = 209 (71.6 %)	With depressive symptoms (SDS ≥ 50) *n* = 83 (28.4 %)	*t*-test or Chi-square	*P* value
	n (%) or Mean (SD)			
Age (y)	24.47 (2.67)	23.31 (2.54)	3.380	0.001
Weeks of pregnancy	34.75 (2.49)	34.60 (2.36)	0.466	0.641
No. of miscarriages	0.66 (0.64)	0.95 (0.89)	-3.144	0.002
Education levels (y)	10.93 (2.29)	10.53 (2.42)	1.331	0.184
Medical expenses			4.544	0.103
Medicaid insurance	136 (65.1)	43 (51.8)		
Self-payment	73 (34.9)	40 (48.2)		
Maternal health status			4.775	0.029
Good	192 (91.8)	69 (83.1)		
General	17 (8.2)	14 (16.9)		
Menstruation history			10.133	0.001
Regular	174 (83.2)	55 (66.2)		
Irregular	35 (16.8)	28 (33.8)		
Antenatal health check			0.850	0.356
Regular	199 (95.2)	81 (97.5)		
Irregular	10 (4.8)	2 (2.5)		
Fetus health worries			3.560	0.059
Yes	180 (86.1)	78 (93.9)		
No	29 (13.9)	5 (6.1)		
Partner relationship			5.706	0.017
Good	162 (77.5)	53 (63.8)		
General	47 (22.5)	30 (10.3)		
Desired fetus sex worries			0.320	0.572
Yes	25 (11.9)	8 (9.6)		
No	184 (88.1)	75 (90.4)		
Preparedness of delivery			12.738	<0.001
Yes	168 (80.3)	50 (60.2)		
No	41 (19.7)	33 (39.8)		
Financial strain			7.071	0.008
Yes	34 (16.2)	25 (30.1)		
No	175 (83.8)	58 (69.9)		
Pregnancy-related stress (total PPS score)	13.62 (9.49)	19.09 (10.94)	-4.250	<0.001
Anxiety (total SAS score)	30.55 (6.05)	34.22 (7.34)	-4.385	<0.001
Coping styles by SCSQ				
Active coping	1.65 (0.82)	1.30 (0.55)	3.524	<0.001
Passive coping	1.00 (0.48)	0.96 (0.48)	0.793	0.429
Social support (total SSRS score)	42.50 (14.92)	38.66 (6.71)	2.253	0.025

*Abbreviations: PPS* Pregnancy Pressure Scale, *SCSQ* Simplified Coping Style Questionnaire, *SAS* Self-rating Anxiety Scale, *SDS* Self-rating Depression Scale, *SSRS* Social Support Rating Scale

Table 2 summarizes the estimates of standardized direct, indirect and total effects of socio-demographic factors, pregnancy-related stress, social support and coping strategies on depressive symptoms. The stressors of financial strain (0.177) and pregnancy-related stress (0.211) have both direct and indirect effects on depressive symptoms. Mediators of total social support (-0.113) and active coping (-0.144) have direct effects on depressive symptom scores. Anxiety symptoms (0.192) only have a direct effect on depressive symptoms. A path model was established, as shown in Fig. 2. Standard beta weights were used to represent path coefficients, as presented on each arrow in Fig. 2. All of the path coefficients are significant at the level of *P* < 0.05. This path model yielded a good fit for stressors, mediators of depressive and anxiety symptoms among Chinese primipara (χ^2^ = 32.317, p = 0.061, CFI = 0.961, TLI = 0.917, IFI = 0.964, NFI = 0.900, RMSEA = 0.042).

**Table 2:** Summary of the direct, indirect, and total effects of significant factors on depressive symptoms among Chinese primipara

Variables	Effects	Significant factors									
		No. of miscarriage	Partner relationship	Irregular menstruation history	Financial strain	Preparedness for delivery	Pregnancy-related stress	Social support	Active coping	Passive coping	Anxiety symptoms
Depressive symptoms	Direct	0.000	0.000	0.000	0.144	-0.144	0.144	-0.113	-0.144	0.000	0.192
	Indirect	0.097	-0.051	0.027	0.033	0.000	0.067	0.000	-0.044	0.047	0.000
	Total	0.097	-0.051	0.027	0.177	-0.144	0.211	-0.113	-0.188	0.047	0.192

## Discussion

A conceptual model of antenatal stressors, mediators and depressive symptoms has been established among Chinese primipara. This path model supported the proposed model within the theoretical framework of the stress process model [21]. Pregnancy-related stress, financial strain and active coping have both direct and indirect effects on depressive symptoms. In line with previous research [25, 31], active coping was a significant mediator between socio-demographic risk factors and antenatal depressive symptoms. High levels of stress would activate a pregnant woman’s coping resources and reduce the impairment of her subsequent well-being [32]. In this study, when women experience higher levels of pregnancy-related stress and/or other socio-demographic stressors, such as financial strain, their personal resources for coping are triggered. Thus, acting out coping strategies would decrease depressive symptoms.

Psychological preparedness for delivery, social support and anxiety levels have direct effects on antenatal depressive symptoms. Good preparedness for delivery could reduce depressive symptoms, while higher levels of anxiety could significantly increase depressive symptoms. Hence, relevant antenatal education interventions are needed to implement for enhancing pregnant women’s psychological preparedness for delivery. The components of antenatal education interventions have to be including prenatal maternal stress management and relevant active coping strategies with stress, given the indirect effect of active coping with reducing women’s depressive symptoms in this study. The format and timing of interventions can be face-to-face in outpatient clinics of obstetrics department or be offered by web-based, which could be decide by pregnant women’s preferences.

Consistent with previous research [33], social support has direct mediating effects on socio-demographic factors and stress outcomes of depressive symptoms. Other research also confirmed that pregnant women who receive social support could be protected from stressful events [34]. The current findings were also in accordance with psychosocial stress theories, that social support is a positive mediator for antenatal depressive and anxiety symptoms [18, 35]. Additionally, the indirect effects of miscarriage history, irregular menstruation, partner relationship and passive coping with depressive symptom levels suggest that poor quality of partner relationship, miscarriage and irregular menstruation history were possible additional stressors, while passive coping was negative personal resources when dealing with depressive symptoms.

This path model was established in a cross-sectional study design rather than a prospective longitudinal design, so that causal inference or directions between study variables can be precluded. Further research is needed to conduct a long-term follow-up of the subjects in order to test this model as longitudinally robust, and relationships between study variables as predictive. In addition, study subjects were recruited from a maternal and child hospital. The subjects, who were mainly in their 20s, were made up only of Chinese primipara, so that generalization of these results is limited by the homogeneity of the study sample. Future research should be conducted using a multicentered approach, and recruit multiparous women. This predictive correlational study only used secondary analysis of data. Further qualitative assessments could complement the limitations of this study approach. Another limitation is that some scales such as SAS and SDS were not designed to be used during pregnancy. Pregnancy is a special condition in the woman’s life, which certainly interferes with values. Therefore, there is a need for future research to develop specific scales for women during pregnancy to screen their anxiety and depressive symptoms.

Despite these limitations, however, the study offers implications for antenatal care practice. Study results have implications for antenatal care practice, with the ultimate aim of reducing depressive symptoms among pregnant women. As pregnancy-related stress has a direct effect on increased incidence of antenatal depressive and anxiety symptoms, relevant service interventions should be developed to eliminate those sources of stressors, in order to enhance the well-being of pregnant women. In addition, an antenatal outreach program should be developed in order to promote the well-being of pregnant women and improve existing antenatal care in China. As active coping and social support during the antenatal period appear to be major factors related to the psychological health of pregnant women [16, 25], training pregnant women’s active coping strategies and promoting social support are further directions for enhancement of antenatal service delivery.

## Conclusion

This study has tested and established a pertinent conceptual model of stressor, mediators and depressive symptoms within the conceptual framework of stress-process-model. This path model has enriched theories on the determinants of depressive symptoms among Chinese primipara. The empirical support from this study could facilitate the formulation of appropriate interventions for reducing antenatal depressive symptoms, and enhancing the mental health of pregnant women.
